# Treatment switching in evidence synthesis in oncology: A systematic review of current meta-analytical practices

**DOI:** 10.1017/rsm.2026.10076

**Published:** 2026-03-09

**Authors:** Rebecca Kathleen Metcalfe, Quang Vuong, Yichen Yan, Anders Gorst-Rasmussen, Antonio Remiro-Azócar, Antonia Morga, Oliver Keene, Louis Dron, Jay J.H. Park

**Affiliations:** 1 Core Clinical Sciences Inc., Vancouver, BC, Canada; 2 Centre for Advancing Health Outcomes, The University of British Columbia, Vancouver, BC, Canada; 3 Department of Statistics & Actuarial Science, Simon Fraser University, Burnaby, BC, Canada; 4 HTA Data Science, Novo Nordisk A/S, Denmark; 5 Methods and Outreach, Novo Nordisk, Spain; 6 Global HEOR, Astellas Pharma Ltd, UK; 7 Statistics, KeeneONStatistics, UK; 8 Redwood AI, Vancouver, Canada; 9 Department of Health Research Methods, Evidence and Impact, McMaster University, Hamilton, ON, Canada

**Keywords:** bias, Estimands, evidence synthesis, meta-analysis, post-randomization events, treatment switching

## Abstract

The 2019 ICH E9(R1) addendum highlights the importance of estimands, including the specification of post-randomization events that may affect the interpretation of clinical trial outcomes (i.e., intercurrent events; ICEs) and strategies to handle these events. Compared to trial protocols, there is limited discussion of estimands in the context of evidence synthesis. We conducted a comprehensive review of the Cochrane Library for pairwise meta-analyses of immuno-, targeted, hormone, and other novel oncology therapies. Dates were restricted to 2021 and onwards to allow time for addendum adoption. Outcomes of interest were progression-free survival (PFS) and overall survival (OS). Information on treatment switching and analytic strategies to address treatment switching were extracted from each meta-analysis and the RCTs they included. Out of 162 oncology reviews published in the Cochrane Library since 2021, eight pairwise meta-analyses and 68 RCTs met selection criteria. Most RCTs were Phase 3 (68%; *n* = 46) and/or open-label (76%; *n* = 52). More than half of RCTs explicitly allowed switching (59%; *n* = 40), while more than one third (38%; *n* = 26) did not report on treatment switching. Among trials that allowed treatment switching, censoring mechanisms for treatment switching varied in analyses of PFS. No included RCTs censored OS at the time of treatment switching. Despite the high prevalence of treatment switching in included trials, none of the identified meta-analyses addressed treatment switching analytically. Poor reporting regarding treatment switching in the RCTs themselves hinders the utility of aggregate-level meta-analyses. To ensure accurate interpretation of meta-analytic results, improved reporting of ICEs and ICE handling strategies is needed.

## Highlights

### What is already known?

The ICH E9(R1) addendum emphasizes the importance of specifying target estimands, including intercurrent events and strategies for their handling, in clinical trials, and highlights that pooling estimates for different target estimands in meta-analysis could be misleading.

### What is new?

We conducted a systematic literature review of meta-analyses in the Cochrane Library and the clinical trials they synthesized to assess current meta-analytic practices related to intercurrent events broadly, and treatment switching specifically. Meta-analyses reviewed pooled estimates employing differing censoring strategies, potentially reflecting differing estimands, without consideration to how this may impact the pooled estimates. Despite most trials allowing treatment crossover, no meta-analyses reviewed addressed treatment switching analytically.

### Potential impact for RSM readers

Current best practices for meta-analysis do not address differences in target estimands from clinical trials. This may introduce bias across pooled estimates relative to the research question of interest in the meta-analysis. New methods are needed to accommodate heterogeneity in target estimands in meta-analysis.

## 1 Introduction

Systematic reviews and meta-analyses form the backbone of treatment guidelines worldwide.^1^^–^ ^5^ These evidence synthesis techniques typically rely on findings from randomized clinical trials (RCTs). Unfortunately, the interpretation and applicability of the treatment effects reported by RCTs is often unclear.^6^^,^ ^7^ This problem is particularly evident when considering the effect of intercurrent events (also referred to as post-randomization events) on interpretation of trial results. For example, in oncology trials, interpretation of overall survival may differ based on how the common intercurrent event of treatment switching is handled analytically. To reduce ambiguity in the interpretation of clinical trial results, the International Council for Harmonisation of Technical Requirements for Pharmaceuticals for Human Use (ICH) in 2019 approved the E9(R1) addendum adopting the estimands framework.^8^ The addendum was motivated by a lack of clarity in the handling of intercurrent events. For instance, many analyses reported as “intention-to-treat” (ITT) “ignored” intercurrent events by stopping to follow up participants after the intercurrent event. In the case of treatment switching, the “ITT” analyses would inadvertently estimate treatment effects in hypothetical settings where treatment switching does not take place by censoring at time of treatment switching. However, this implies excluding outcome data for subjects experiencing the intercurrent event, thereby diverging from the ITT principle, which should include follow-up measurements for all subjects under their randomized treatment allocation, regardless of adherence to the planned course of treatment.

The estimands framework outlines the necessary elements to unambiguously operationalize a clinical research question and to align the trial objectives, design and analytic approaches with such question. The operationalized question is called an estimand. Once an estimand has been defined, suitable methods of estimation (“estimators”) can be selected, and numerical results (“estimates”) can be obtained.^9^ A core component of an estimand is the specification of likely intercurrent events affecting interpretation of the outcome and the strategies for handling them.

The estimands framework is explicit in outlining five strategies, including the treatment policy strategy, which aligns with the ITT principle and includes any effect resulting from the occurrence of the intercurrent event in the definition of the treatment effect, and the hypothetical strategy, which envisages a scenario in which the intercurrent event does not occur. In the case of treatment switching, the treatment policy estimand includes follow-up time on the investigational treatment as well as subsequent therapies and might be of most clinical interest if all subsequent therapies in the trial are approved and reliable. However, if none of the subsequent therapies in the trial are approved, the hypothetical strategy that envisions a hypothetical scenario where the subsequent therapies are not available may be of most clinical interest.^10^

Following publication of the ICH E9(R1) addendum, peer-reviewed articles have addressed the importance of estimands for clinical trial planning and interpretation^11^ and have assessed the quality of estimand reporting in RCTs and RCT protocols published in leading journals.^6^^,^ ^12^ However, limited attention has been paid to the impact of the estimands framework, and specifically intercurrent events, on evidence synthesis. Given the large influence of meta-analyses on clinical treatment guidelines around the world, and the impact of intercurrent events on the practical meaning of trial results, inadequate attention to intercurrent events in meta-analyses could have far-reaching consequences. Recognizing this, the ICH E9(R1) addendum explicitly notes that integration of data from multiple trials without consideration and specification of the estimand targeted by each trial, which includes handling of intercurrent events, can be misleading.^8^ Yet to date, no guidance exists on how to address intercurrent events in evidence synthesis.

**Figure 1:**
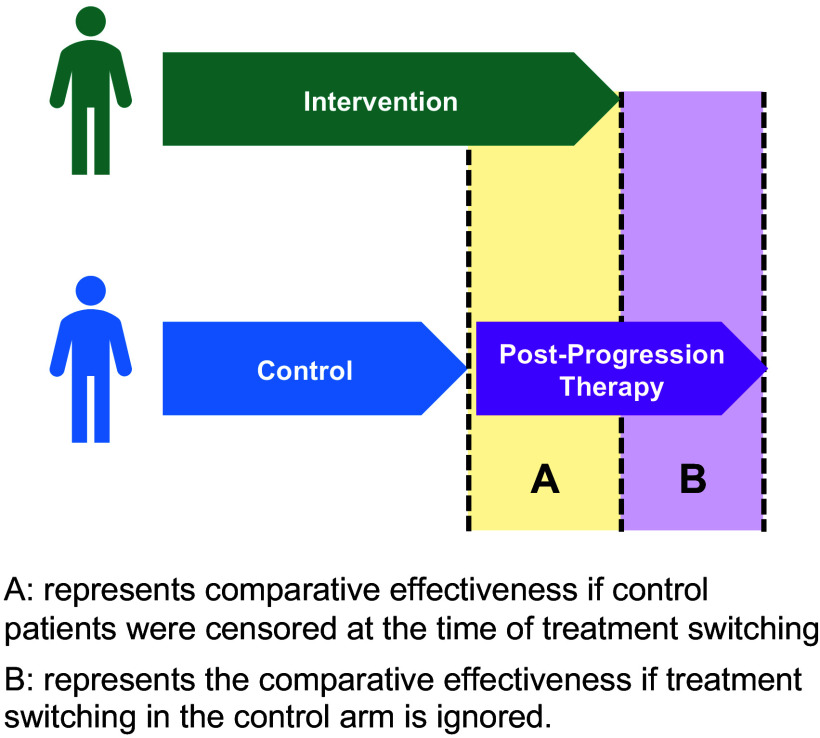
Example of impact of treatment switching on trial estimates.

Thus, the purpose of this systematic literature review was to assess current methods for handling intercurrent events in evidence synthesis. Specifically, this review focused on practices for treatment switching because it is a common and well-known intercurrent event in oncology (Figure 1). ^10^^,^ ^13^ Indeed, the National Institute for Health and Care Excellence (NICE) has released two guidance documents to address treatment switching in clinical trials.^14^^,^ ^15^ It should be noted that for ethical reasons, treatment switching is often built into the trial protocol for oncology trials and hence does not represent a protocol violation. The review examined treatment switching in oncology meta-analyses published in the Cochrane Library, since it is widely recognized as the gold standard for the synthesis of medical evidence.^16^^–^ ^18^ In addition to assessing how treatment switching was handled in the meta-analyses themselves, this review investigated reporting of treatment switching in the individual RCTs that the meta-analyses synthesized.

## 2 Methods

This systematic literature review was designed in accordance with the Preferred Reporting Items for Systematic Reviews and Meta-Analysis (PRISMA) guidelines.^19^ The review was registered with *PROSPERO* (CRD42023487365) prior to commencing data collection.^20^

### 2.1 Data sources and searches

Meta-analyses published in the Cochrane Library that addressed immune-, targeted, hormone, and other novel oncology therapies were reviewed. Dates were restricted to 2021 and onwards to allow time for adoption of the ICH E9(R1) addendum. Outcomes of interest were progression-free survival (PFS) and overall survival (OS). Information on treatment switching and analytical strategies to account for treatment switching were extracted from each meta-analysis and the RCTs they included. The search strategy included different word variations of cancer (specifically: “oncology,” “cancer,” and “neoplasm”) using the “Title, Abstract, and Keyword” filter.

### 2.2 Study inclusion and exclusion criteria

Complete study eligibility criteria are described in Table 1. In brief, this review included peer-reviewed Cochrane reviews reporting on pairwise meta-analyses of randomized clinical trials in oncology. Interventions of interest were novel therapies defined as those approved 10 years or fewer before the review inclusion window (i.e., in 2011 or after) or yet to be approved. Chemotherapies, radiotherapies, and surgical interventions were excluded. There were no restrictions on the comparators. Reviews were eligible if they reported on at least one outcome between PFS and OS. Any definition or timeframe of assessment for PFS or OS was permitted.

**Table 1:** PICOS criteria Table 1 Long description.

Category	Criteria
Population	Patients with any cancer site or class, and of any stage or treatment setting
Intervention	Novel oncological treatments defined as those approved 10 years or fewer before the review inclusion window (i.e., in 2011 or after) or yet to be approved Chemotherapies, radiotherapies, and surgical interventions were excluded
Comparators	No restrictions
Outcomes	At least one of the following outcomes: Progression-free survival Overall survival
Study design	Meta-analyses and network meta-analyses of randomized clinical trials identified through a systematic review
Other	Published in Cochrane Library in or after 2021

Two reviewers (RKM and JJHP) independently reviewed all abstracts and proceedings identified in the literature searches. The full text publications of potentially relevant abstracts were then retrieved and assessed for eligibility. Two reviewers (RKM and JJHP) also screened the bibliographies of published Cochrane reviews for further eligible Cochrane reviews potentially missed by the search strategy. Discrepancies in study selection were resolved through a discussion between the two reviewers or, when necessary, by a third investigator (LD). After identifying the eligible systematic reviews with meta-analyses, their bibliographies were hand-searched to obtain the individual clinical trials they included and their respective trial registries (RM, LD, and JJHP). When a Cochrane review included multiple publications for a given trial, data were extracted from the primary publication indicated in the Cochrane review. If no primary publication was indicated or the primary publication was an abstract, the first full-text publication of the primary trial analysis was reviewed. For all included trials, the statistical analysis plan, trial protocol, and trial registration were also reviewed where available.

### 2.3 Data extraction

Study design elements, patient characteristics, and outcomes were extracted independently by two investigators (RKM and JJHP) using a standardized, piloted data extraction form. For Cochrane Reviews, information was recorded on the study characteristics, eligibility criteria, tumor and biomarker types, intervention types, outcomes (PFS and/or OS), consideration of any intercurrent event and treatment switching, specifically, in risk of bias assessment, and stated strategies for handling treatment switching in the evidence synthesis. For individual clinical trials, information was recorded on tumor and biomarker type, number of study arms, therapy details, sample size, trial registry, reported outcome definitions of PFS and OS and their related censoring mechanism, analytical set (in this case, per protocol versus intention-to-treat), and treatment switching, including conditions for treatment switching, treatment crossover, and direction of treatment crossover. Here, we use “treatment switching” to refer to any change from the treatment assigned at randomization to another treatment. We use “treatment crossover” to refer to a specific type of treatment switching in which a participant changes from the treatment assigned at randomization to another treatment under investigation in the study. Discrepancies were resolved through a discussion between the two reviewers.

### 2.4 Data synthesis

A meta-analysis was not conducted for this study. The findings of this review are presented descriptively. First, the findings from the meta-analyses are described, followed by the characteristics of the trials they synthesized.

### 2.5 Role of the funding source

This study was not funded.

## 3 Results

### 3.1 Evidence base

**Figure 2:**
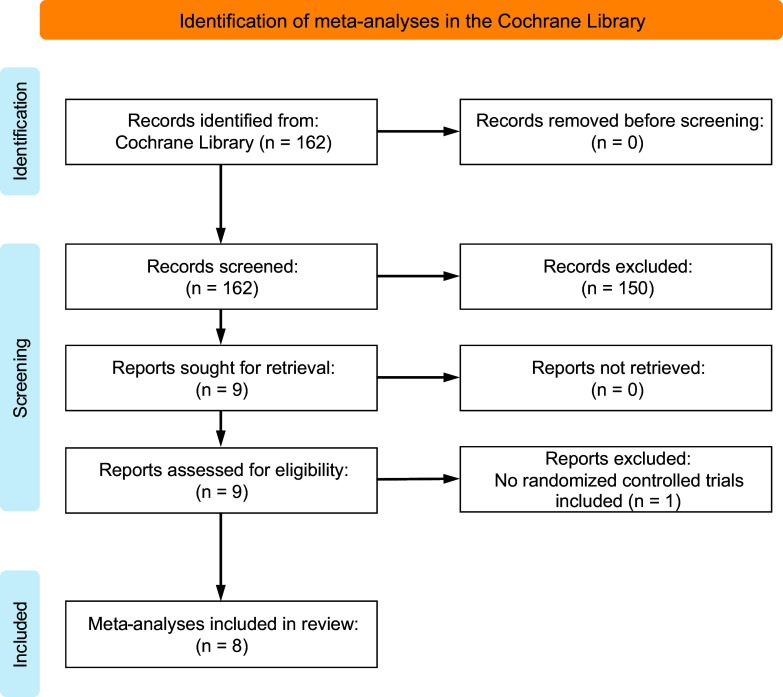
PRISMA diagram for meta-analysis search. Figure 2 Long description.

**Figure 3:**
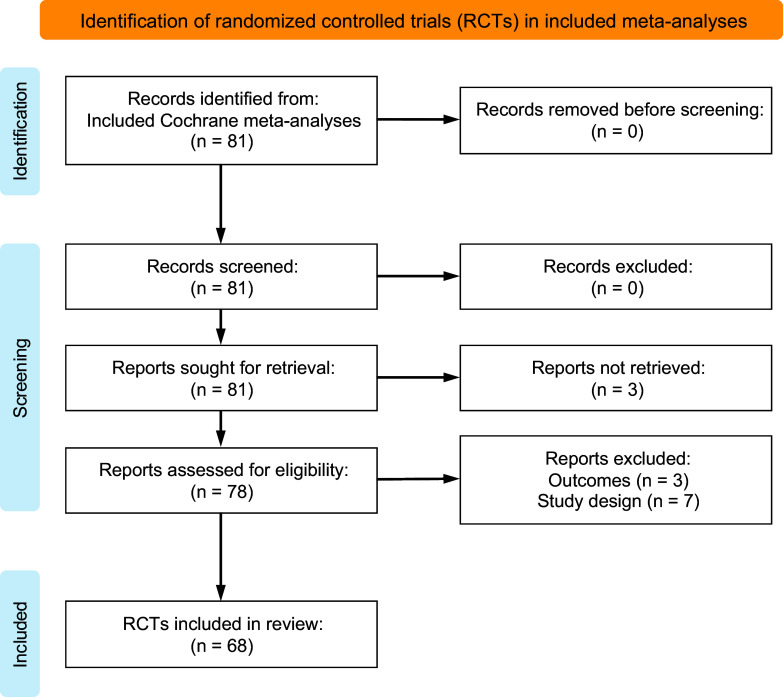
PRISMA diagram for randomized controlled trial search. Figure 3 Long description.

The search of the Cochrane Library identified 162 reviews of which eight were eligible meta-analyses (see Figure 2). ^21^^–^ ^28^ These eight eligible Cochrane meta-analyses included 81 unique clinical trials. Of these, 68 were eligible for inclusion in the review (see Figure 3; see Supplementary Table 9 for more detail on reasons for trial exclusion).

Of the eight Cochrane reviews, four meta-analyses (*n* = 4) addressed novel therapeutics for lung cancer, and the other four addressed breast cancer (*n* = 1), melanoma (*n* = 1), cervical cancer (*n* = 1), and urothelial carcinoma (*n* = 1). None of these reviews mentioned estimands and intercurrent events or post-randomization events. One meta-analysis considered treatment impact on OS only, while the remaining seven assessed impact on both OS and PFS (see Table 2 for details).

**Table 2:** Summary of cochrane meta-analyses reviewed Table 2 Long description.

Lead				Intercurrent	Data analysis
author of				events considered	accounting
Cochrane		Clinical	Outcomes	in risk-of-bias	for intercurrent
Review	Year	context	studied	assessment?	events?
Gorry	2023	Melanoma	PFS and OS	No	No
Cameron	2022	Lung cancer	PFS and OS	No	Report median length of survival time with confidence intervals as well as crossover rate. No additional analyses performed.
Taylor	2021	Breast cancer	PFS and OS	Yes—in the context of formal crossover trials	Planned sensitivity analysis for studies that allowed crossover, but hazard ratios accounting for crossover were not available, so no formal sensitivity analysis was conducted.
Greenhalgh	2021	Lung cancer	PFS and OS	No	No
Zhu	2021	Lung cancer	PFS and OS	No	If formal crossover studies had been included, planned to use data from first phase (i.e., randomization to crossover) only.
Chuai	2021	Cervical cancer	OS	No	No
Ferrara	2021	Lung cancer	PFS and OS	Yes—considered intercurrent illness in the notes of study characteristics for one included trial (Reck, 2016)	If formal crossover studies had been included, planned to follow Cochrane Handbook recommendations for formal crossover studies.
Maisch	2023	Urothelial carcinoma	PFS and OS	No	No

PFS: progression-free survival; OS: overall survival; RCT: randomized controlled trial.

Of the 68 unique RCTs eligible for this review, 68% were Phase 3 (*n* = 46) and 76% were open-label (*n* = 52) (Table 3). Most trials reported both PFS and OS (85%; *n* = 58), with a smaller number reporting only OS (10%; *n* = 7) or PFS (3%; *n* = 3). Most studies conducted primary analyses for PFS and OS using the intention-to-treat analysis set (81%; *n* = 55). However, 15% of studies (*n* = 10) did not explicitly report the analysis set.

**Table 3:** Summary of trials reviewed Table 3 Long description.

Trial characteristic	% (*n*)
Reported trial phase	
Phase 1^a^	1% (1)
Phase 1/2^a^	1% (1)
Phase 2^a^	22% (15)
Phase 3	68% (46)
Not reported	7% (5)
Blinding	
Blinded	15% (10)
Open-label	76% (52)
Other	1% (1)
Not reported	7% (5)
Treatment switching	
Allowed	59% (40)
Not allowed	3% (2)
Not reported	38% (26)
Direction of treatment switching^b^ (*n* = 40)	
Crossover from control to intervention	59% (23)
Crossover from intervention to control	41% (16)
Switch to another therapy not studied in the trial	46% (18)
Not reported	13% (5)
Reasons for treatment switching^b^ (*n* = 40)	
Disease progression	85% (35)
Safety/tolerability	40% (16)
Clinician discretion	30% (12)
Patient preference	18% (7)
Other	10% (4)
No reason reported	10% (4)

a Trials all involved randomization

b Numbers do not sum to 100% as multiple options were possible for a given trial

### 3.2 Evaluation of meta-analyses

#### 3.2.1 Assessment of intercurrent events in included randomized clinical trials

The Cochrane risk-of-bias tool for randomized trials (RoB 2) is the recommended tool to assess risk-of-bias in randomized trials included in Cochrane Reviews.^29^^,^ ^30^ Of the eight eligible Cochrane meta-analyses, two considered intercurrent events in their risk-of-bias assessment using RoB 2. The first study, Taylor et al. (2021), considered the impact of intercurrent events, specifically treatment crossover, on estimates of OS.^25^ They assessed five criteria pertaining to treatment switching in their RoB 2 assessment. These criteria focused on randomization, differences between trial arms in baseline characteristics, frequency of censoring events, analysis set (per protocol versus intention-to-treat), and follow-up schedules. The second study, Ferrara et al.^26^ considered intercurrent events in their RoB 2 assessment for one included trial, where they noted that developing an illness with another condition could introduce bias into the study analysis. No other meta-analysis RoB 2 assessments included intercurrent events broadly or treatment switching specifically.

#### 3.2.2 Analytic approaches to treatment switching

Most (*n* = 6) of the included meta-analyses did not report any analytic strategy for addressing treatment switching. Among the two meta-analyses that reported strategies, planned strategies varied and were not tied to a specific outcome. Cameron et al.^27^ reported the median length of survival time as well as the crossover rate within each trial to help contextualize findings but did not conduct a formal analysis. Taylor et al.^25^ planned sensitivity analyses addressing crossover in studies in which crossover was allowed but the required hazard ratios were not available for the sensitivity analysis to be conducted.

### 3.3 Evaluation of included randomized clinical trials

#### 3.3.1 Treatment switching

Of the 68 eligible RCTs, the majority allowed some form of treatment switching (59%; *n* = 40) (Table 3). A substantial proportion of trials did not report explicitly whether treatment switching was permitted (38%; *n* = 26). Only two (3%) of the RCTs reviewed stated that treatment switching was not allowed. Among the 40 trials that stated that treatment switching was allowed, reasons for treatment switching varied. Almost all trials explicitly allowed treatment switching following disease progression (85%; *n* = 35). The next most common reason for treatment switching was safety/tolerability (40%; *n* = 16). Four (10%) of the trials that allowed treatment switching did not report any reason for treatment switching. Only one study (*n* = 3%) reported the timing of treatment switching.

Among those RCTs that allowed treatment switching, most (70%; *n* = 28/40) allowed crossover between trial arms. Eleven studies (28%) allowed participants to crossover both from control to intervention and from intervention to control. Twelve studies (30%) only allowed crossover from control to intervention, and five studies (13%) only allowed crossover from intervention to control.

Of the 28 studies which stated that crossover was allowed, all but one reported the rate of crossover between trial arms (96%; *n* = 27).

##### 3.3.1.1 Outcome definition

Across studies, similar definitions of PFS and OS were reported in primary publications. In general, PFS was defined as the time from randomization to first disease progression or death. Disease progression was often assessed per the RECIST (Response Evaluation Criteria In Solid Tumors) criteria.^31^ OS was defined as the time from randomization to death due to any cause. Censoring rules related to treatment switching that inform the definition of PFS and OS (e.g., censoring PFS at the time of treatment switching) were often not reported in primary publications.

##### 3.3.1.2 Analytic approaches to address treatment switching

As censoring rules related to treatment switching were infrequently reported in the primary publications, analysis of censoring in PFS and OS was limited to trials for which at least one of a statistical analysis plan or trial protocol was publicly available (43%; *n* = 29/68). Of these 29 trials, one trial reported on OS only, while the rest reported on PFS and OS. Censoring rules for PFS and OS differed. More than half of trials (54%; *n* = 15/28) censored participants from the primary PFS analysis for reasons related to treatment switching, while roughly one third (36%; *n* = 10/28) did not. It was not possible to determine censoring rules for PFS related to treatment switching for the remaining trials (11%; *n* = 3). Of the 15 trials that censored participants at the time of treatment switching, only four (27%) reported censoring rules related to treatment switching in the primary study report.^32^^–^ ^35^ The main text detail provided varied across these four trials. One trial did not describe the censoring mechanism directly but stated that censoring rules were described in the Supplementary Material .^32^ A separate trial described the censoring mechanism in the primary publication but the description differed from that in the accompanying protocol.^35^ In contrast to the diverse censoring strategies adopted for PFS, no trials censored OS at the time of treatment switching.

Censoring approaches varied between trials analyzed within a single meta-analysis. For example, the trials by Bellmunt et al.^35^ and Galsky et al.^36^ were both included in the same meta-analysis^23^ but employed different censoring approaches pertaining to treatment switching in their respective primary PFS analyses. While Bellmunt et al. included participants who switched treatments before progression in the primary PFS analysis, Galsky et al. censored them. The treatment effect estimates were pooled in the meta-analysis regardless of this difference.

Outside of censoring in the primary analyses, statistical analysis plans and protocols reported ancillary (i.e., secondary or exploratory) analyses to understand the impact of treatment switching on efficacy estimates. These were more commonly planned for PFS than for OS (48%, *n* = 14/28; and 35%, *n* = 10/29, respectively). In general, ancillary analyses consisted of sensitivity analyses where the primary analysis was repeated using different censoring rules. For example, Powles et al. (2020) did not censor participants at the time of treatment switching in their primary analysis of PFS.^32^ However, to better understand how non-trial treatments may have impacted efficacy estimates, the study protocol included a sensitivity analysis where participants were censored at the last evaluation prior to switching treatments. Less commonly, investigators planned more specialized analyses of treatment switching. For example, in addition to repeating the analysis with different censoring rules, the statistical analysis plan of Camidge et al. (2020) included a re-analysis of OS using rank preserving structural failure time modeling (RPSFTM) and, separately, inverse probability of censoring weights.^37^^,^ ^38^

## 4 Discussion

This review found that despite treatment switching being a common intercurrent event in oncology trials, a minority of the eight Cochrane meta-analyses reviewed reported any strategy for addressing treatment switching, and more than half did not consider treatment switching at all. The limited attention paid to this common intercurrent event may be due to generally poor trial reporting of treatment switching. Of the 68 trials included in the Cochrane meta-analyses and reviewed here, more than one third did not report on treatment switching at all. This absence is concerning given the potential implications of treatment switching, and particularly treatment crossover, on the interpretation of efficacy estimates. When trials use different censoring rules for treatment switching, they may be addressing or aligning with different clinical questions. For instance, analyses that censor patients at the time of switching target a hypothetical estimand, while those that include follow-up after switching without adjustment reflect a treatment-policy estimand. Pooling these estimates in a single meta-analysis combines results that are not strictly comparable, which can obscure the clinical meaning of the pooled treatment effect.^39^ Notably, although most studies that allowed crossover reported the rate of within-study crossover, only one reported timing of crossover, something recognized as crucial for accurate interpretation.^40^

The limited reporting on censoring rules relating to the intercurrent event of treatment switching, an intercurrent event that is well known to directly impact the interpretation of time-to-event outcomes and treatment effects for PFS and OS, is worrying. In the minority of eligible trials that had a publicly available protocol or statistical analysis plan, there was substantial variation in censoring rules for PFS, which was commonly used as the primary trial outcome. Roughly half (*n* = 15/28) of trials censored participants from the primary PFS analysis when treatment switching occurred before disease progression and most of these studies did not report censoring of PFS at treatment switching in the text of the primary publication.

This review highlights challenges in interpreting the results of clinical trials and meta-analyses that label the trial analyses as “intention-to-treat” or “per protocol.” While both terms may have a clear meaning at a high-level, their operationalization tends to differ between trials.^41^ These differences can be meaningful for interpretation of study results but are currently obscured by traditional terminology. For example, Sezer and colleagues (2021) reported OS and PFS as co-primary endpoints analyzed following the intention-to-treat principle.^42^ However, the principle was operationalized differently for each endpoint: the primary analysis of OS included all participants regardless of treatment switching; in contrast, the primary analysis of PFS censored participants at the time of treatment switching. As a result, the primary analyses of this single trial have different interpretations.

Similarly, the Cochrane meta-analyses reviewed stated that they prioritized intention-to-treat analyses for meta-analysis of PFS and OS, with no discussion of the operationalization of intention-to-treat across outcomes and across trials. It is unclear how to interpret the results of meta-analyses that pool effect estimates without consideration to varying handling of intercurrent events. Speaking to this issue, the Grading of Recommendations Assessment, Development and Evaluation (GRADE) working group developed strategies for evidence synthesis that address treatment switching in RCTs.^43^ Here, the working group recommends that trial methods be evaluated for alignment with the primary research aim of the evidence synthesis. In cases where misalignment exists (e.g., the trial reports on a hypothetical estimand when the evidence synthesis is focused on a treatment policy estimand), the strength of evidence rating for the trial should be downgraded for indirectness. This represents an important step forward toward more transparent evidence synthesis in the context of treatment switching but does not directly tackle the issue of including treatment effects that target different estimands in a single meta-analysis.^10^^,^ ^44^

High-quality meta-analyses based on well-conducted RCTs are widely considered the most robust form of clinical evidence.^45^ As a result, they underpin key decisions by actors across the care spectrum—from health technology assessment bodies that determine which treatments will be added to regional formularies to clinicians whose care decisions are shaped by clinical guidelines.^1^^,^ ^46^ Thus, ambiguity in interpreting the results of meta-analyses may have wide-reaching consequences. At present, systematic reviews and meta-analyses often use the PICO (population, intervention, comparator, and outcome) framework to specify their research questions.^47^^–^ ^49^ However, the PICO framework may not be optimal for meta-analyses because it allows room for ambiguity around intercurrent events, like treatment switching, which may impact the interpretation of trial results.^50^ In contrast, the estimands framework adopted by the ICH E9(R1) addendum requires specification of intercurrent events and strategies to handle them, making the scope of trial estimates clear. Meta-analysis could benefit from the estimands framework by pre-specifying a target estimand and making explicit how different intercurrent events present in included trials would be handled. Adopting the estimands framework for meta-analysis would not eliminate ambiguity, particularly in complex clinical contexts like treatment switching in oncology, but it would help delineate the relevance of the derived effect estimates to different clinical questions of interest. In this way, adoption of the estimands framework for meta-analysis could support decision-makers by making the relevance of evidence clearer, particularly regarding intercurrent events such as treatment switching.^50^ This perspective also matters for stakeholders that translate evidence into guidance, such as Cochrane, NICE, and the World Health Organization. Clarifying the target estimand within systematic reviews would help ensure that pooled estimates truly reflect the treatment effect relevant to policy and clinical decision-making.

### 4.1 Strengths and limitations

This systematic literature review benefits from the use of PRISMA guidelines and registration in the *PROSPERO* database prior to commencement. By focusing on Cochrane meta-analyses, the gold standard for evidence synthesis in health research, the insights gained from this review are likely to be applicable to other meta-analyses that have been conducted with less oversight and following less rigorous guidelines. To contextualize current meta-analytic methods in the context of intercurrent events, this review considered available supporting RCT documentation, such as trial protocols and statistical analysis plans.

However, this review should be considered in the context of its limitations. While every effort was made to comprehensively evaluate the included publications, trial registries, and Supplementary Material, protocols and statistical analysis plans were only available for fewer than half of included RCTs (43%; *n* = 29/68). Furthermore, our review was limited to treatment switching in oncology. We chose treatment switching specifically because it is well established as an important and common intercurrent event in oncology trials. Thus, if meta-analyses are not considering this prominent intercurrent event, it is unlikely that they would account for other important intercurrent events such as use of rescue medication. Nevertheless, more work is needed to assess practices in other clinical contexts and therapeutic areas. Additionally, our focus on the meta-analytical practices of published Cochrane reviews likely resulted in reviews performed by academic researchers. While our review allowed more than one year between the official release of the ICH E9(R1) addendum and the publication of eligible meta-analyses, that may not have been sufficient time for meta-analytic studies to adapt to the estimands framework. As the ICH E9(R1) addendum was developed as a collaboration between regulators and the life sciences industry, it is largely directed to industry-sponsored trials for regulatory submission. It is possible that an assessment of reviews sponsored or performed by the pharmaceutical industry may have resulted in different findings. However, a 2020 review of published trials and industry submissions to NICE by Sullivan et al. (2020) found similarly inadequate reporting of treatment switching.^40^

### 4.2 Future directions

This review clearly indicates a need for more consistent and comprehensive clinical trial reporting. Nearly half of the trials with available protocols or statistical analysis plans included ancillary analyses pertaining to treatment switching. Thus, there is broad awareness that treatment switching and related censoring strategies impact efficacy estimates. Indeed, there have been recent calls to improve reporting and consideration of subsequent therapies in oncology trials.^51^^,^ ^52^ In tandem, investigators should report informative censoring strategies as well as ancillary analyses of related estimands as part of the primary trial publication, as recommended by the ICH E9(R1) addendum.^8^ In practical terms, future systematic reviews could pre-specify which estimand they intend to target and can be inferred from each included trial (e.g., based on the choice of analysis method), how intercurrent events were handled. Data-extraction templates could include dedicated fields for intercurrent events and their analytical strategies. Sensitivity analyses could then explore how pooled results change when restricted to trials sharing a similar estimand or when adjusting for different handling approaches. Publication of ancillary analyses may also enable the investigation of more specific research questions in meta-analysis, as investigators could access data targeting multiple estimands from each trial. Furthermore, it is a common meta-analytical practice to digitize published Kaplan–Meier curves to create pseudo-individual patient-level data for meta-analyses in the time-to-event setting. In terms of trial reporting, reporting Kaplan–Meier curves for primary and ancillary estimands with varying strategies for treatment switching and other intercurrent events could enable more robust evidence synthesis. In the absence of improved reporting, guidance is needed on assessing estimand concordance for the purpose of meta-analysis and methods for evaluating potential bias resulting from pooling different estimands.

The finding that estimates derived using different censoring or model-based strategies for treatment switching are pooled in meta-analyses of oncology trials warrants further consideration as these analytic differences may correspond to differing target estimands. As the ICH E9(R1) addendum warns, pooling of different estimands can result in misleading estimates of treatment effects. Future studies should consider exploring what bias (relative to the target estimand) may be introduced by pooling treatment effect estimates targeting different estimands in evidence synthesis and meta-analysis.

The estimands framework offers an opportunity to define the target of estimation for meta-analysis to improve the specificity and relevance of study findings to decision-makers. However, there is a balance to be considered between the specificity of the question considered in a meta-analysis and the breadth of information available to answer it. More specific research questions likely improve relevance to specific decision-making contexts, but give rise to narrower study inclusion criteria, which may reduce the feasibility of meta-analysis due to a scarcity of trials. A related consequence could be disconnected evidence networks even in network meta-analysis. As no one estimand will ever be the only estimand of interest, new meta-analytic methods are needed that can accommodate pooling of multiple estimands in order to make best use of all available scientific evidence. More specifically, research should target how to align meta-analytic estimates with the clinical estimands of interests in the context of heterogeneity in clinical trial estimands. Adaptations of existing hierarchical or model-based methods, such as multilevel network meta-regression or model-based network meta-analysis, may be fruitful.^53^^,^ ^54^

In the absence of these methods, broader awareness of the importance of intercurrent events for evidence synthesis is needed to better reflect clinical evidence. There are currently no estimand guidelines targeting academic trialists or institutions like Cochrane. Integration of estimands into existing trial reporting guidelines would be an important step forward. Although the most recent CONSORT guidelines could not reach consensus on the inclusion of estimands,^55^^,^ ^56^ several CONSORT extensions and other trial reporting guidelines already recommend the estimands framework.^57^^–^ ^61^ The need for specificity in efficacy estimates derived from clinicals trials and meta-analysis is not limited to industry settings as evidenced by the recent GRADE working group guidelines on treatment switching.^43^ Taken together this points to the need for further work to ensure that the same transparency standards will be implemented for both industry and non-industry sponsored trials.

## Supporting information

10.1017/rsm.2026.10076.sm001 Metcalfe et al. supplementary material Metcalfe et al. supplementary material

## Data Availability

The dataset compiled for this systematic literature review is available from the corresponding author upon reasonable request.

## Supplementary material

To view supplementary material for this article, please visit http://doi.org/10.1017/rsm.2026.10076.

## Long descriptions

### Table 1 Long description

The table consists of two columns: Category and Criteria.

Row 1: Population. Criteria includes patients with any cancer site or class, and of any stage or treatment setting.

Row 2: Intervention. Criteria includes novel oncological treatments defined as those approved 10 years or fewer before the review inclusion window (i.e., in 2011 or after) or yet to be approved. Chemotherapies, radiotherapies, and surgical interventions were excluded.

Row 3: Comparators. No restrictions.

Row 4: Outcomes. At least one of the following outcomes: Progression-free survival or Overall survival.

Row 5: Study design. Meta-analyses and network meta-analyses of randomized clinical trials identified through a systematic review.

Row 6: Other. Published in Cochrane Library in or after 2021.

Navigate back to Table 1.

### Figure 2 Long description

The flowchart is organized into three vertical phases labeled on the left: Identification, Screening, and Included.

1. Identification Phase

- Records identified from Cochrane Library n equals 162. An arrow points right to Records removed before screening n equals 0. An arrow points down to the next phase.

2. Screening Phase

- Records screened n equals 162. An arrow points right to Records excluded n equals 150. An arrow points down.

- Reports sought for retrieval n equals 9. An arrow points right to Reports not retrieved n equals 0. An arrow points down.

- Reports assessed for eligibility n equals 9. An arrow points right to Reports excluded because no randomized controlled trials were included n equals 1. An arrow points down to the final phase.

3. Included Phase

- Meta-analyses included in review n equals 8.

Navigate back to Figure 2.

### Figure 3 Long description

The flowchart is divided into three vertical phases.

1. Identification phase.

At the top left, a box indicates Records identified from Included Cochrane meta-analyses n equals 81. An arrow points right to Records removed before screening n equals 0. A downward arrow leads to the next phase.

2. Screening phase.

This phase contains four sequential steps.

First, Records screened n equals 81, with an arrow pointing right to Records excluded n equals 0.

Second, Reports sought for retrieval n equals 81, with an arrow pointing right to Reports not retrieved n equals 3.

Third, Reports assessed for eligibility n equals 78, with an arrow pointing right to a box listing Reports excluded due to Outcomes n equals 3 and Study design n equals 7.

A downward arrow leads to the final phase.

3. Included phase.

The final box at the bottom center states R C T s included in review n equals 68.

Navigate back to Figure 3.

### Table 2 Long description

The table consists of six columns and eight rows of data.

* Row 1: Gorry (2023), Melanoma, P F S and O S, No, No.

* Row 2: Cameron (2022), Lung cancer, P F S and O S, No, Report median length of survival time with confidence intervals as well as crossover rate. No additional analyses performed.

* Row 3: Taylor (2021), Breast cancer, P F S and O S, Yes—in the context of formal crossover trials, Planned sensitivity analysis for studies that allowed crossover, but hazard ratios accounting for crossover were not available, so no formal sensitivity analysis was conducted.

* Row 4: Greenhalgh (2021), Lung cancer, P F S and O S, No, No.

* Row 5: Zhu (2021), Lung cancer, P F S and O S, No, If formal crossover studies had been included, planned to use data from first phase (i.e., randomization to crossover) only.

* Row 6: Chuai (2021), Cervical cancer, O S, No, No.

* Row 7: Ferrara (2021), Lung cancer, P F S and O S, Yes—considered intercurrent illness in the notes of study characteristics for one included trial (Reck, 2016), If formal crossover studies had been included, planned to follow Cochrane Handbook recommendations for formal crossover studies.

* Row 8: Maisch (2023), Urothelial carcinoma, P F S and O S, No, No.

Abbreviations used: P F S stands for progression-free survival and O S stands for overall survival.

Navigate back to Table 2.

### Table 3 Long description

The table consists of two columns: Trial characteristic and percent (n).

* Reported trial phase: Phase 1 is 1 percent (1); Phase 1/2 is 1 percent (1); Phase 2 is 22 percent (15); Phase 3 is 68 percent (46); Not reported is 7 percent (5).

* Blinding: Blinded is 15 percent (10); Open-label is 76 percent (52); Other is 1 percent (1); Not reported is 7 percent (5).

* Treatment switching: Allowed is 59 percent (40); Not allowed is 3 percent (2); Not reported is 38 percent (26).

* Direction of treatment switching (n equals 40): Crossover from control to intervention is 59 percent (23); Crossover from intervention to control is 41 percent (16); Switch to another therapy not studied in the trial is 46 percent (18); Not reported is 13 percent (5).

* Reasons for treatment switching (n equals 40): Disease progression is 85 percent (35); Safety/tolerability is 40 percent (16); Clinician discretion is 30 percent (12); Patient preference is 18 percent (7); Other is 10 percent (4); No reason reported is 10 percent (4).

Navigate back to Table 3.
