# Supplementary material for: Treatment switching in evidence synthesis in oncology: A systematic review of current meta-analytical practices
Source: Res Synth Methods. 2026 Mar 9;17(4):782–96. doi: 10.1017/rsm.2026.10076 (PMC13311355; doi:10.1017/rsm.2026.10076)
Supplement: Metcalfe et al. supplementary material [file S1759287926100763sup001.docx]

**Supplementary Materials:**

Supplement to “Treatment Switching in Evidence Synthesis in Oncology: A Systematic Review of Current Meta-Analytical Practices

# Supplementary Tables

Supplementary Table 1: Included randomized controlled trials from Gorry C, McCullagh L, O'Donnell H, et al. Neoadjuvant treatment for stage iii and iv cutaneous melanoma. Cochrane Database of Systematic Reviews. 2023;(1)

| **Trial ID** | **Trial Reference** |
| --- | --- |
| 1-Amaria 2018a-1 | Amaria RN, Prieto PA, Tetzlaff MT, Reuben A, Andrews MC, Wargo JA, et al. Neoadjuvant plus adjuvant dabrafenib and trametinib versus standard of care in patients with high-risk, surgically resectable melanoma: a single-centre, open-label, randomised, phase 2 trial. *Lancet Oncology* 2018;19(2):181-93. |
| 1-Amaria 2018b-1 | Amaria RN, Reddy SM, Tawbi HA, Davies MA, Ross MI, Glitza IC, et al. Neoadjuvant immune checkpoint blockade in high-risk resectable melanoma. *Nature Medicine* 2018;24(11):1649-54. |
| 1-Blank 2018-1 | Blank CU, Rozeman EA, Fanchi LF, Sikirska K, van de Wiel B, Schumacher TN, et al. Neoadjuvant versus adjuvant ipilimumab plus nivolumab in macroscopic stage III melanoma. *Nature Medicine* 2018;24:1655-61. [NCT02437279] |
| 1-Dummer 2020b-11 | Dummer R, Gyorki DE, Hyngstrom JR, Ning M, Lawrence T, Ross MI. Final 5-Year Follow-Up Results Evaluating Neoadjuvant Talimogene Laherparepvec Plus Surgery in Advanced Melanoma: A Randomized Clinical Trial. JAMA oncology. 2023 Oct 1;9(10):1457-9. |

Supplementary Table 2: Included randomized controlled trials from Cameron LB, Hitchen N, Chandran E, et al. Targeted therapy for advanced anaplastic lymphoma kinase (alk)‐rearranged non‐small cell lung cancer. Cochrane Database of Systematic Reviews. 2022;(1)

| **Study ID** | **Study Reference** |
| --- | --- |
| 2-Alesia 2019-1 | Zhou C, Kim SW, Reungwetwattana T, Zhou J, Zhang Y, He J, et al. Alectinib versus crizotinib in untreated Asian patients with anaplastic lymphoma kinase-positive non-small-cell lung cancer (ALESIA): a randomised phase 3 study. *Lancet* 2019;7(5):437-46. [DOI: 10.1016/S2213-2600(19)30053-0] |
| 2-Alex 2017-1 | Peters S, Camidge DR, Shaw AT, Gadgeel S, Ahn JS, Kim DW, et al. Alectinib versus crizotinib in untreated ALK-positive non-small-cell lung cancer. *New England Journal of Medicine* 2017;377(9):829-38. [DOI: 10.1056/NEJMoa1704795] |
| 2-ALTA-1L-2019-1 | Camidge D R, Kim HR, Ahn MJ, Yang JCH, HanJY, Hochmair MJ, et al. Brigatinib versus crizotinib in advanced ALK inhibitor-naive ALK-positive non-small cell lung cancer: second interim analysis of the phase III ALTA-1L trial. Journal of Clinical Oncology 2020;38(31):3592-603. [DOI: 10.1200/JCO.20.00505] |
| 2-ALUR 2018-10 | Wolf J, Helland Å, Oh IJ, Migliorino MR, Dziadziuszko R, Wrona A, de Castro J, Mazieres J, Griesinger F, Chlistalla M, Cardona A. Final efficacy and safety data, and exploratory molecular profiling from the phase III ALUR study of alectinib versus chemotherapy in crizotinib-pretreated ALK-positive non-small-cell lung cancer. ESMO open. 2022 Feb 1;7(1):100333. |
| 2-ASCEND-4 2017-4 | Soria JC, Tan DSW, Chiari R, Wu YL, Paz-Ares L, Wolf J, et al.First-line ceritinib versus platinum-based chemotherapy in advanced ALK-rearranged non-small-cell lung cancer (ASCEND-4): a randomised, open-label, phase 3 study. *Lancet* 2017;389(10072):917-29. [DOI: 10.1016/ S0140-6736%2817%2930123-X] |
| 2-ASCEND-5 2017-1 | Shaw AT, Kim TM, Crinò L, Gridelli C, Kiura K, Liu G, et al.Ceritinib versus chemotherapy in patients with ALK- rearranged non-small-cell lung cancer previously given chemotherapy and crizotinib (ASCEND-5): a randomised, controlled, open-label, phase 3 trial. *Lancet Oncology* 2017;18(7):874-86. [DOI: 10.1016/S1470-2045(17)30339-X] |
| 2-CROWN 2020-1 | Shaw A, Bauer T, Takahashi T, Baik C, Goto Y, Polli A, et al.First-line lorlatinib versus crizotinib for advanced anaplastic lymphoma kinase-positive (ALK+) non-small cell lung cancer. *Journal of Thoracic Oncology* 2018;13(10):S584. [DOI: 10.1016/ j.jtho.2018.08.863] |
| 2-J-ALEX 2017-1 | Hida T, Nokihara H, Kondo M, Kim YH, Azuma K, Seto T, et al.Alectinib versus crizotinib in patients with ALK-positive non- small-cell lung cancer (J-ALEX): an open-label, randomised phase 3 trial. Lancet 2017;390(10089):29-39. [DOI: 10.1016/ S0140-6736(17)30565-2] |
| 2-PROFILE 1007 2013-1 | Shaw AT, Kim DW, Nakagawa K, Seto T, Crinó L, Ahn MJ, et al.Crizotinib versus chemotherapy in advanced ALK- positive lung cancer. New England Journal of Medicine 2013;368(25):2385-94. [DOI: 10.1056/NEJMoa1214886] |
| 2-PROFILE 1014 2014-1 | Solomon BJ, Mok T, Kim DW, Wu YL, Nakagawa K, Mekhail T, et al.First-line crizotinib versus chemotherapy in ALK-positive lung cancer. New England Journal of Medicine 2014;371(23):2167-77. [DOI: 10.1056/NEJMoa1408440] |
| 2-PROFILE 1029 2018-1 | Wu YL, Lu S, Lu Y, Zhou J, Shi YK, Sriuranpong V, et al.Results of PROFILE 1029, a phase III comparison of first-line crizotinib versus chemotherapy in East Asian patients with ALK-positive advanced non–small cell lung cancer. Journal of Thoracic Oncology 2018;13(10):1539-48. [DOI: 10.1016/j.jtho.2018.06.012] |

Supplementary Table 3: Included randomized controlled trials from Taylor AM, Chan DLH, Tio M, et al. Parp (poly adp‐ribose polymerase) inhibitors for locally advanced or metastatic breast cancer. Cochrane Database of Systematic Reviews. 2021;(4)

| **Study ID** | **Study Reference** |
| --- | --- |
| 3-BROCADE 2-1 | Han HS, Diéras V, Robson M, Palácová M, Marcom PK, Jager A et al. Veliparib with temozolomide or carboplatin/ paclitaxel versus placebo with carboplatin/paclitaxel in patients with BRCA1/2 locally recurrent/metastatic breast cancer: randomized phase II study. Annals of Oncology 2018;29(1):154-61. |
| 3-BROCADE 3-1 | Dieras V, Han H, Kaufman B, Wildiers H, Friedlander M, Ayoub J, et al. A Phase 3 randomized, placebo-controlled trial of carboplatin and paclitaxel with or without the PARP inhibitor veliparib (ABT-888) in HER2-negative metastatic or locally advanced unresectable BRCA-associated breast cancer. Annals of Oncology 2019;30(Suppl 5):v851-v934. |
| 3-EMBRACA-1 | Litton JK, Rugo HS, Ettl J, Hurvitz SA, Gonçalves A, Lee KH, et al. Talazoparib in patients with advanced breast cancer and a germline BRCA mutation. New England Journal of Medicine 2018;379(8):753-63. |
| 3-Kummar 2016-1 | Kummar S, Wade JL, Oza AM, Sullivan D, Chen AP, Gandara DR, et al. Randomized phase II trial of cyclophosphamide and the oral poly (ADP-ribose) polymerase inhibitor veliparib in patients with recurrent, advanced triple-negative breast cancer. Investigational New Drugs 2016;34(3):355–63. |
| 3-OLYMPIAD-1 | Robson M, Im SA, Senkus E, Xu B, Domchek SM, Masuda N, et al. Olaparib for metastatic breast cancer in patients with a germline BRCA mutation. New England Journal of Medicine 2017;377(6):523-33. |

Supplementary Table 4: Included randomized controlled trials from Greenhalgh J, Boland A, Bates V, et al. First‐line treatment of advanced epidermal growth factor receptor (egfr) mutation positive non‐squamous non‐small cell lung cancer. Cochrane Database of Systematic Reviews. 2021;(3)

| **Study ID** | **Study Reference** |
| --- | --- |
| 4-BMSO99 -1 | Lynch TJ, Patel T, Dreisbach L, McCleod M, Heim WJ, Hermann RC, et al. Cetuximab and first-line taxane/carboplatin chemotherapy in advanced non-small cell lung cancer: results of the randomized multicenter phase III trial BMS099. *Journal of Clinical Oncology* 2010;28(6):911-7. |
| 4-CHEN-1 | Chen YM, Tsai CM, Fan WC, Shih JF, Liu SH, Wu CH, et al. Phase II randomized trial of erlotinib or vinorelbine in chemonaive, advanced, non-small cell lung cancer patients aged 70 years or older. *Journal of Thoracic Oncology* 2012;7(2):412-8. |
| 4-CONVINCE-1 | Shi YK, Wang L, Han BH, Li W, Yu P, Liu YP, et al. First-line icotinib versus cisplatin/pemetrexed plus pemetrexed maintenance therapy for patients with advanced EGFR mutation-positive lung adenocarcinoma (CONVINCE): a phase 3, open-label, randomized study. *Annals of Oncology* 2017;28(10):2443-50. |
| 4-ENSURE-1 | Wu Y-L, Zhou C, Liam CK, Wu G, Liu X, Zhong Z, et al. First- line erlotinib versus gemcitabine/cisplatin in patients with advanced EGFR mutation-positive non-small-cell lung cancer: analyses from the phase III, randomized, open-label, ENSURE study. *Annals of Oncology* 2015;26(9):1883-9. |
| 4-EURTAC-1 | Rosell R, Carcereny E, Gervais R, Vergnenegre A, Massuti B, Felip E, et al. Erlotinib versus standard chemotherapy as first- line treatment for European patients with advanced EGFR mutation-positive non-small-cell lung cancer (EURTAC): a multicentre, open-label, randomised phase 3 trial. *Lancet Oncology* 2012;13(3):239-46. |
| 4-FASTACT 2-1 | Wu YL, Lee JS, Thongprasert S, Yu CJ, Zhang L, Ladrera G, et al. Intercalated combination of chemotherapy and erlotinib for patients with advanced stage non-small-cell lung cancer (FASTACT-2): a randomised, double-blind trial. *Lancet Oncology* 2013;14(8):777-86. |
| 4-First-SIGNAL-1 | Han JY, Park K, Kim SW, Lee DH, Kim HY, Kim HT, et al. First- SIGNAL: first-line single-agent Iressa versus gemcitabine and cisplatin trial in never-smokers with adenocarcinoma of the lung. *Journal of Clinical Oncology* 2012;30(10):1122-8. |
| 4-FLEX-2 | Pirker R, Pereira JR, Szczesna A, Von Pawel J, Krzakowski M, Ramlau R, et al. Cetuximab plus chemotherapy in patients with advanced non-small-cell lung cancer (FLEX): an open-label randomised phase III trial. *Lancet* 2009;373(9674):1525-31. |
| 4-INTACT 1-1 | Giaccone G, Herbst R, Manegold C, Scagliotti G, Rosell R, Miller V, et al. Gefitinib in combination with gemcitabine and cisplatin in advanced non-small cell lung cancer: a phase III trial - INTACT 1. *Journal of Clinical Oncology* 2004;22:777-84. |
| 4-IPASS-1 | Mok TS, Wu YL, Thongprasert S, Yang CH, Chu DT, Saijo N, et al. Gefitinib or carboplatin-paclitaxel in pulmonary adenocarcinoma. *New England Journal of Medicine* 2009;361(10):947-57. |
| 4-LUX-Lung 3-1 | Sequist LV, Yang JCH, Yamamoto N, O’Byrne K, Hirsh V, Mok T, et al. Phase III study of afatinib or cisplatin plus pemetrexed in patients with metastatic lung adenocarcinoma with EGFR mutations. *Journal of Clinical Oncology* 2013;31:1-11. |
| 4-LUX-Lung 6-1 | Wu Y-L, Zhou C, Hu C-P, Feng J, Lu S, Huang Y, et al. Afatinib versus cisplatin plus gemcitabine for first-line treatment of Asian patients with advanced non-small-cell lung cancer harbouring EGFR mutations (LUX-Lung 6): an open-label, randomised phase 3 trial. *Lancet Oncology* 2014;15(2):213-22. |
| 4-NEJSG-1 | Maemondo M, Inoue A, Kobayashi K, Sugawara S, Oizumi S, Isobe H, et al. Gefitinib or chemotherapy for non–small cell lung cancer with mutated EGFR. *New England Journal of Medicine* 2010;362(25):2380-8. |
| 4-OPTIMAL-1 | Zhou C, Wu YL, Chen G, Feng J, Liu XQ, Wang C, et al. Erlotinib versus chemotherapy as first-line treatment for patients with advanced EGFR mutation positive non-small cell lung cancer (OPTIMAL, CTONG-0802): a multicentre, open-label, randomised, phase 3 study. Lancet Oncology 2011;12(8):735-42. |
| 4-TORCH-1 | Gridelli C, Ciardiello F, Gallo C, Feld R, Butts C, Gebbia V, et al. First-line erlotinib followed by second-line cisplatin- gemcitabine chemotherapy in advanced non-small cell lung cancer: the TORCH randomized trial. *Journal of Clinical Oncology* 2012;30(24):3002-11. |
| 4-WJTOG3405-1 | Mitsudomi T, Morita S, Yatabe Y, Negoro S, Okamoto I, Tsurutani J, et al. Gefitinib versus cisplatin plus docetaxel in patients with non-small cell lung cancer harbouring mutations of the epidermal growth factor receptor (WJTOG3405): an open label, randomised phase 3 trial. Lancet Oncology 2009;11(2):121-8. |
| 4-Yu 2014-1 | Yu H, Zhang J, Wu X, Luo Z, Wang H, Sun S, et al. A phase II randomized trial evaluating gefitinib intercalated with pemetrexed/platinum chemotherapy or pemetrexed/platinum chemotherapy alone in unselected patients with advanced non- squamous non-small cell lung cancer. *Cancer Biology & Therapy* 2014;15(7):832-9. |

Supplementary Table 5: Included randomized controlled trials from Zhu J, Yuan Y, Wan X, et al. Immunotherapy (excluding checkpoint inhibitors) for stage to iii non‐small cell lung cancer treated with surgery or radiotherapy with curative intent. *Cochrane Database of Systematic Reviews*. 2021;(12)

| **Study ID** | **Study Reference** |
| --- | --- |
| 5-Butts 2014-1 | Butts C, Socinski MA, Mitchell PL, Thatcher N, Havel L, Krzakowski M, et al. Tecemotide (L-BLP25) versus placebo after chemoradiotherapy for stage III non-small-cell lung cancer(START): a randomised, double-blind, phase 3 trial. *Lancet Oncolology* 2014;15(1):59-68. |
| 5-Fujisawa 1996-1 | Fujisawa T, Yamaguchi Y. Postoperative immunostimulation after complete resection improves survival of patients with stage I non-small cell lung carcinoma. *Cancer* 1996;78(9):1892-8. |
| 5-Giovanni 1996-1 | Giovanni BR, Paolo Z, Sandro M, Paolo M, Riccardo A, Giovanni F, et al. A randomized trial of adoptive immunotherapy with tumor-infiltrating lymphocytes and interleukin-2 versus standard therapy in the postoperative treatment of resected non-small cell lung carcinoma. *Cancer* 1996;78(2):244-51. |
| 5-Katakami 2017-1 | Katakami N, Hida T, Nokihara H, Imamura F, Sakai H, Atagi S, et al. Phase I/II study of tecemotide as immunotherapy in Japanese patients with unresectable stage III non-small cell lung cancer. *Lung Cancer* 2017;105:23-30. |
| 5-Macchiarini 1991-1 | Macchiarini P, Hardin M, Angeletti CA. Long-term evaluation of intrapleural Bacillus Calmette-Guérin with or without adjuvant chemotherapy in completely resected stages II and III non- small-cell lung cancer. *American Journal of Clinical Oncology* 1991;14(4):291-7. |
| 5-Matthay 1986-1 | Matthay RA, Mahler DA, Beck GJ, Loke J, Baue AE, Carter DC, et al. Intratumoral Bacillus Calmette-Guérin immunotherapy prior to surgery for carcinoma of the lung: results of a prospective randomized trial. *Cancer Research* 1986;46(11):5963-8. |
| 5-Multhoff 2020-1 | Multhoff G, Seier S, Stangl S, Sievert W, Shevtsov M, Werner C, et al. Targeted natural killer cell-based adoptive immunotherapy for the treatment of patients with NSCLC after radiochemotherapy: a randomized phase II clinical trial. *Clinical Trials: Immunotherapy* 2020;26:5368-79. |
| 5-Stanley 1986-1 | Stanley K, Ludwig Lung Cancer Study Group (LLCSG). Immunostimulation with intrapleural BCG as adjuvant therapy in resected non-small cell lung cancer. *Cancer* 1986;58(11):2411-6. |
| 5-Vansteenkiste 2013-1 | Vansteenkiste J, Zielinski M, Linder A, Dahabreh J, Gonzalez EE, Malinowski W, et al. Adjuvant MAGE-A3 immunotherapy in resected non-small-cell lung cancer: phase II randomized study results. *Journal of Clinical Oncology* 2013;31(19):2396-403. |
| 5-Vansteenkiste 2016-1 | Vansteenkiste JF, Cho BC, Vanakesa T, de Pas T, Zielinski M, Kim MS, et al. Efficacy of the MAGE-A3 cancer immunotherapeutic as adjuvant therapy in patients with resected MAGE-A3-positive non-small-cell lung cancer (MAGRIT): a randomised, double-blind, placebo-controlled, phase 3 trial. *Lancet Oncology* 2016;17(6):822-35. |
| 5-Zhao 2014-1 | Zhao M, Li H, Li L, Zhang Y. Effects of a gemcitabine plus platinum regimen combined with a dendritic cell-cytokine induced killer immunotherapy on recurrence and survival rate of non-small cell lung cancer patients. *Experimental and Therapeutic Medicine* 2014;7(5):1403-7. |

Supplementary Table 6: Included randomized controlled trials from Chuai Y, Rizzuto I, Xia Z, et al. Vascular endothelial growth factor (vegf) targeting therapy for persistent, recurrent, or metastatic cervical cancer. *Cochrane Database of Systematic Reviews*. 2021;(3)

| **Study ID** | **Study Reference** |
| --- | --- |
| 6-Guo 2020-1 | Guo Q, Sun Y, Kong E, Rao L, Chen J, Wu Q, et al. Apatinib combined with chemotherapy or concurrent chemobrachytherapy in patients with recurrent or advanced cervical cancer: a phase 2, randomized controlled, prospective study. *Medicine* 2020;99(11):e19372. |
| 6-Monk 2010-1 | Monk BJ, Mas Lopez L, Zarba JJ, Oaknin A, Tarpin C, Termrungruanglert W, et al. Phase II, open-label study of pazopanib or lapatinib monotherapy compared with pazopanib plus lapatinib combination therapy in patients with advanced and recurrent cervical cancer. *Journal of Clinical Oncology* 2010;28(22):3562-9. |
| 6-Symonds 2015-1 | Symonds RP, Gourley C, Davidson S, Carty K, McCartney E, Rai D, et al. Cediranib combined with carboplatin and paclitaxel in patients with metastatic or recurrent cervical cancer (CIRCCa): a randomised, double-blind, placebo-controlled phase 2 trial. *Lancet Oncology* 2015;16(15):1515-24. |
| 6-Tewari 2014-1 | Tewari KS, Sill MW, Long HJ 3rd, Penson RT, Huang H, Ramondetta LM, et al. Improved survival with bevacizumab in advanced cervical cancer. *New England Journal of Medicine* 2014;370(8):734-43. |

Supplementary Table 7: Included randomized controlled trials from Ferrara R, Imbimbo M, Malouf R, et al. Single or combined immune checkpoint inhibitors compared to first‐line platinum‐based chemotherapy with or without bevacizumab for people with advanced non‐small cell lung cancer. Cochrane Database of Systematic Reviews. 2021;(4)doi:10.1002/14651858.CD013257.pub3

| **Study ID** | **Study Reference** |
| --- | --- |
| 7-Carbone 2017-1 | Carbone DP, Reck M, Paz-Ares L, Creelan B, Horn L, Steins M. First-line nivolumab in stage IV or recurrent non-small-cell lung cancer. *New England Journal of Medicine* 2017;376:2415-26. |
| 7-Hellmann 2018-1 | Hellmann MD, Ciuleanu TE, Pluzanski A, Lee JS, Otterson GA, Audigier-Valette C, et al. Nivolumab plus ipilimumab in lung cancer with a high tumor mutational burden. *New England Journal of Medicine* 2018 May 31;378(22):2093-104. [DOI: 10.1056/NEJMoa1801946] |
| 7-Herbst 2020-1 | Herbst RS, Giaccone G, de Marinis F, Reinmuth N, Vergnenegre A, Barrios CH, et al. Atezolizumab for first-line treatment of PD-L1-selected patients with NSCLC. *New England Journal of Medicine* Oct 2020;383(14):1328-39. |
| 7-Mok 2019-1 | Mok TS, Wu YL, Kudaba I, Kowalski DM, Cho BC, Turna HZ, et al. Pembrolizumab versus chemotherapy for previously untreated, PD-L1-expressing, locally advanced or metastatic non-small-cell lung cancer (KEYNOTE-042): a randomised, open- label, controlled, phase 3 trial. *Lancet* 2019;393(10183):1819-30. |
| 7-Reck 2016-1 | Reck M, Rodríguez-Abreu D, Robinson AG, Hui R, Csőszi T, Fülöp A, et al. KEYNOTE-024 Investigators. pembrolizumab versus chemotherapy for PD-L1-Positive non-small-cell lung cancer. *New England Journal of Medicine* 2016;375(19):1823-33. |
| 7-Rizvi 2020-1 | Rizvi NA, Cho BC, Reinmuth N, Lee KH, Luft A, Ahn MJ, et al. Durvalumab with or without tremelimumab vs standard chemotherapy in first-line treatment of metastatic non- small cell lung cancer: the MYSTIC Phase 3 randomized clinical trial. *JAMA Oncology* 2020;Apr 9:E1-14. [DOI: 10.1001/ jamaoncol.2020.0237] |
| 7-Sezer 2020-2 | NCT03088540. A global, randomised, phase 3, open-label study of REGN2810 (ANTI-PD 1 Antibody) versus platinum based chemotherapy in first line treatment of patients with advanced or metastatic PD L1+non-small cell lung cancer. https://clinicaltrials.gov/ct2/show/NCT03088540 accessed January 2020. |
| 7-Sezer 2020-3 | Sezer A, Kilickap S, Gümüş M, Bondarenko I, Özgüroğlu M, Gogishvili M, Turk HM, Cicin I, Bentsion D, Gladkov O, Clingan P. Cemiplimab monotherapy for first-line treatment of advanced non-small-cell lung cancer with PD-L1 of at least 50%: a multicentre, open-label, global, phase 3, randomised, controlled trial. The Lancet. 2021 Feb 13;397(10274):592-604. |

Supplementary Table 8: Included randomized controlled trials from Maisch P, Hwang EC, Kim K, et al. Immunotherapy for advanced or metastatic urothelial carcinoma. *Cochrane Database of Systematic Reviews*. 2023;(10)

| **Study ID** | **Study Reference** |
| --- | --- |
| 8-Bellmunt 2017-1 | Bellmunt J, de Wit R, Vaughn DJ, Fradet Y, Lee JL, Fong L, et al. Pembrolizumab as second-line therapy for advanced urothelial carcinoma. *New England Journal of Medicine* 2017;376(11):1015-26. [DOI: 10.1056/NEJMoa1613683] |
| 8-Galsky 2020-1 | Galsky MD, Arija JÁ, Bamias A, Davis ID, De Santis M, Kikuchi E, et al. Atezolizumab with or without chemotherapy in metastatic urothelial cancer (IMvigor130): a multicentre, randomised, placebo-controlled phase 3 trial. *Lancet* 2020;16(395):1547-57. [DOI: 10.1016/S0140-6736(20)30230-0] |
| 8-Powles 2017-1 | Powles T, Duran I, van der Heijden MS, Loriot Y, Vogelzang NJ, De Giorgi U, et al. Atezolizumab versus chemotherapy in patients with platinum-treated locally advanced or metastatic urothelial carcinoma (IMvigor211): a multicentre, open-label, phase 3 randomised controlled trial. Lancet 2017;391(10122):748-57. [DOI: 10.1016/s0140-6736(17)33297-x] |
| 8-Powles 2020-1 | Powles T, van der Heijden MS, Castellano D, Galsky MD, Loriot Y, Petrylak DP, et al. Durvalumab alone and durvalumab plus tremelimumab versus chemotherapy in previously untreated patients with unresectable, locally advanced or metastatic urothelial carcinoma (DANUBE): a randomised, open-label, multicentre, phase 3 trial. *2020* Lancet Oncology;21(12):1774-88. [DOI: 10.1016/ S1470-2045(20)30541-6] |
| 8-Powles 2021-1 | Powles T, Csőszi T, Özgüroğlu M, Matsubara N, Géczi L, Cheng SY, et al. Pembrolizumab alone or combined with chemotherapy versus chemotherapy as first-line therapy for advanced urothelial carcinoma (KEYNOTE-361): a randomised, open-label, phase 3 trial. *Lancet Oncology* 2021;22(7):931-45. [DOI: 10.1016/S1470-2045(21)00152-2] |

Supplementary Table 9: Excluded randomized controlled trials

| **Study ID** | **Study Reference** | **Reason For Exclusion** | **Details** |
| --- | --- | --- | --- |
| 1-Albertini 2018-1 | Albertini MR, Yang RK, Ranheim EA, Hank JA, Zuleger CL, Weber S, et al. Pilot trial of the hu14.18-IL2 immunocytokine in patients with completely resectable recurrent stage III or stage IV melanoma. Cancer Immunology Immunotherapy 2018;67(10):1647-58. [DOI: 10.1007/s00262-018-2223-z] | Outcomes | Outcomes only for treatment arm |
| 1-Hwu2017-1 | NCT00525031. Temozolomide alone or with pegylated interferon-alpha 2b (PGI) in melanoma patients [Randomized phase II neoadjuvant study of temozolomide alone or with pegylated interferon-alpha 2b in patients with resectable American Joint Committee on Cancer (AJCC) stage IIIB/IIIC or stage IV (M1a) metastatic melanoma]. clinicaltrials.gov/ct2/ show/nct00525031 (first received 5 September 2007). | Other | No publication or SAP |
| 1-Rozeman 2019-1 | Rozeman EA, Menzies AM, van Akkooi ACJ, Adhikari C, Bierman C, van de Wiel BA, et al. Identification of the optimal combination dosing schedule of neoadjuvant ipilimumab plus nivolumab in macroscopic stage III melanoma (OpACIN-neo): a multicentre, phase 2, randomised, controlled trial. *Lancet Oncology* 2019;20(7):948-60. | Outcomes | No OS reported and recurrence free survival, but not PFS |
| 1-Tarhini 2018-1 | Tarhini A, Lin Y, Lin H, Rahman A, Vallabhaneni P, Mendiratta P, et al. Neoadjuvant ipilimumab (3 mg/kg or 10 mg/kg) and high dose IFN-alpha2b in locally/regionally advanced melanoma: safety, efficacy and impact on T-cell repertoire. *Journal for Immunotherapy of Cancer* 2018;6(1):112. | Outcomes | No PFS/OS |
| 3-BRAVO-1 | Balmana J, Tryfonidis K, Audeh W, Goulioti T, Slaets L, Agarwal S, et al. A phase III, randomized, open-label, multicenter, controlled trial of niraparib versus physician's choice in previously-treated, HER2-negative, germline BRCA mutation-positive breast cancer patients. An EORTC-BIG intergroup study (BRAVO study). Cancer Research 2016;76(Suppl 4):Abstract nr OT1-03-05. | Other | Abstract only |
| 4-GTOWG-1 | Reck M, Von Pawel J, Fischer Jr, Kortsik C, Von Eiff M, Koester W, et al. Erlotinib versus carboplatin/vinorelbine in elderly patients (age 70 or older) with advanced non-small cell lung carcinoma (NSCLC): a randomised phase II study of the German Thoracic Oncology Working Group. *Journal of Clinical Oncology* 2010;28:15s. | Other | Conference abstract only |
| 8-Iacovelli 2022-1 | Iacovelli R, Ciccarese C, Brunelli M, Battelli N, Buttigliero C, Caserta C, et al. First line avelumab in PD-L1+ve metastatic or locally advanced urothelial cancer (aUC) patients unfit for cisplatin (cis): the ARIES trial. Journal of Clinical Oncology 2022;40(6 Suppl):439. | Study Design | Exclude - single arm trial, no randomization |
| 8-Massard 2016-1 | Massard C, Gordon MS, Sharma S, Rafii S, Wainberg ZA, Luke J, et al. Safety and efficacy of durvalumab (MEDI4736), an anti–programmed cell death ligand-1 immune checkpoint inhibitor, in patients with advanced urothelial bladder cancer. Journal of Clinical Oncology 2016;34(26):3119-25. [DOI: 10.1200/ JCO.2016.67.9761] | Study Design | Exclude - single arm trial, no randomization |
| 8-Patel 2017-1 | Patel MR, Ellerton J, Infante JR, Agrawal M, Gordon M, Aljumaily R, et al. Avelumab in metastatic urothelial carcinoma after platinum failure (JAVELIN Solid Tumor): pooled results from two expansion cohorts of an open-label, phase 1 trial. Lancet Oncology 2018;19(1):51-64. [DOI: 10.1016/ S1470-2045(17)30900-2] | Study Design | Exclude - single arm trial, no randomization |
| 8-Sharma 2016-1 | Sharma P, Callahan MK, Bono P, Kim J, Spiliopoulou P, Calvo E, et al. Nivolumab monotherapy in recurrent metastatic urothelial carcinoma (CheckMate 032): a multicentre, open- label, two-stage, multi-arm, phase 1/2 trial. Lancet Oncology 2016;17(11):1590-98. [DOI: 10.1016/S1470-2045(16)30496-X] | Study Design | Exclude - single arm trial, no randomization |
| 8-Sharma 2017-1 | Sharma P, Retz M, Siefker-Radtke A, Baron A, Necchi A, Bedke J, et al. Nivolumab in metastatic urothelial carcinoma after platinum therapy (CheckMate 275): a multicentre, single- arm, phase 2 trial. Lancet Oncology 2017;18(3):312-22. [DOI: 10.1016/s1470-2045(17)30065-7] | Study Design | single-arm design |
| 8-Sheng 2020-1 | Chen H, Sheng X, Zhang R, Hu B, Yao X, Liu Z, et al. Recombinant humanized anti-PD-1 monoclonal antibody toripalimab in patients with refractory/metastatic urothelial carcinoma: preliminary results of an open-label phase II clinical study. European Urology Open Science 2020;19:e1579. [DOI: 10.1016/ S2666-1683(20)33652-1] | Study Design | non-randomized design |
| 8-Ye 2021-1 | Ye D, Liu J, Zhou A, Zou Q, Li H, Fu C, et al. Tislelizumab in Asian patients with previously treated locally advanced or metastatic urothelial carcinoma. Cancer Science 2021;112(1):305-13. [DOI: 10.1111/cas.14681] | Study Design | non-randomized design |
| PFS: Progression-free survival; OS: Overall survival | | | |

Supplementary Table 10: Characteristics of included randomized controlled trials

| **Study ID** | **Trial ID** | **Trial Registration Number** | **Trial Phase** | **Blinding** | **Was treatment switching allowed?** | **Did the trial measure OS or PFS?** |
| --- | --- | --- | --- | --- | --- | --- |
| 1-Amaria 2018a-1 | NCT02231775 | NCT02231775 | Phase 2 | Open-label | Not reported | OS |
| 1-Amaria 2018b-1 | NCT02519322 | NCT02519322 | Phase 2 | Open-label | Not reported | PFS and OS |
| 1-Blank 2018-1 | OpACIN | NCT02437279 | Phase 1 | Open-label | Not reported | OS |
| 1-Dummer 2020b-11 | NCT02211131 | NCT02211131 | Phase 2 | Open-label | No | OS |
| 2-Alesia 2019-1 | NCT02838420 | NCT02838420 | Phase 3 | Open-label | Yes | PFS and OS |
| 2-Alex 2017-1 | ALEX | NCT02075840 | Phase 3 | Open-label | Yes | PFS and OS |
| 2-ALTA-1L-2019-1 | ALTA-1L | NCT02737501 | Phase 3 | Open-label | Yes | PFS and OS |
| 2-ALUR 2018-1 | ALUR | NCT02604342 | Phase 3 | Open-label | Yes | PFS and OS |
| 2-ASCEND-4 2017-4 | ASCEND-4 | NCT01828099 | Phase 3 | Open-label | Yes | PFS and OS |
| 2-ASCEND-5 2017-1 | ASCEND-5 | NCT01828112 | Phase 3 | Open-label | Yes | PFS and OS |
| 2-CROWN 2020-1 | CROWN | NCT03052608 | Phase 3 | Open-label | No | PFS and OS |
| 2-J-ALEX 2017-1 | J-ALEX | JapicCTI-132316 | Phase 3 | Open-label | Yes | PFS and OS |
| 2-PROFILE 1007 2013-1 | PROFILE 1007 | NCT00932893 | Phase 3 | Open-label | Yes | PFS and OS |
| 2-PROFILE 1014 2014-1 | PROFILE 1014 | NCT01154140 | Phase 3 | Open-label | Yes | PFS and OS |
| 2-PROFILE 1029 2018-1 | PROFILE 1029 | NCT01639001 | Phase 3 | Open-label | Yes | PFS and OS |
| 3-BROCADE 2-1 | BROCADE 2 | NCT01506609 | Phase 2 | Blinded | Not reported | PFS and OS |
| 3-BROCADE 3-1 | BROCADE 3 | NCT02163694 | Phase 3 | Blinded | Yes | PFS and OS |
| 3-EMBRACA-1 | EMBRACA | NCT01945775 | Phase 3 | Open-label | Yes | PFS and OS |
| 3-Kummar 2016-1 | NCT01306032 | NCT01306032 | Phase 2 | Open-label | Yes | PFS |
| 3-OLYMPIAD-1 | OlympiAD | NCT02000622 | Phase 3 | Open-label | Yes | PFS and OS |
| 4-BMSO99 -1 | BMS099 | NCT00112294 | Phase 3 | Open-label | Yes | PFS and OS |
| 4-CHEN-1 | Chen 2012 | NR | Phase 2 | Open-label | Not reported | PFS and OS |
| 4-CONVINCE-1 | CONVINCE | NR | Phase 3 | Open-label | Yes | PFS and OS |
| 4-ENSURE-1 | ENSURE | NCT01342965 | Phase 3 | Open-label | Yes | PFS and OS |
| 4-EURTAC-1 | EURTAC | NCT00446225 | Phase 3 | Open-label | Yes | PFS and OS |
| 4-FASTACT 2-1 | FASTACT 2 | NCT00883779 | Phase 3 | Blinded | Yes | PFS and OS |
| 4-First-SIGNAL-1 | First SIGNAL | NCT00455936 | Phase 3 | Open-label | Yes | PFS and OS |
| 4-FLEX-2 | FLEX | NCT00148798 | Phase 3 | Open-label | Yes | PFS and OS |
| 4-Han 2017-1 | NCT02148380 | NCT02148380 | Phase 2 | Open-label | Not reported | PFS and OS |
| 4-INTACT 1-1 | INTACT 1 | NR | Phase 3 | Blinded | Not reported | PFS and OS |
| 4-INTACT 2-1 | INTACT 2 | NR | Phase 2 | Blinded | Not reported | PFS and OS |
| 4-IPASS-1 | IPASS | NCT00322452 | Phase 3 | Open-label | Yes | PFS and OS |
| 4-LUX-Lung 3-1 | LUX Lung 3 | NCT00949650 | Phase 3 | Open-label | Yes | PFS and OS |
| 4-LUX-Lung 6-1 | LUX Lung 6 | NCT01121393 | Phase 3 | Open-label | Yes | PFS and OS |
| 4-NEJSG-1 | NEJSG | UMIN-CTR number C000000376 | Phase 3 | Not reported | Yes | PFS and OS |
| 4-OPTIMAL-1 | OPTIMAL | NCT00874419 | Phase 3 | Open-label | Not reported | PFS and OS |
| 4-Patil 2017-1 | Patil 2017 | CTRI/2015/08/006113 | Phase 3 | Open-label | Yes | PFS and OS |
| 4-TOPICAL-1 | TOPICAL | ISRCTN 77383050 | Phase 3 | Blinded | Not reported | PFS and OS |
| 4-TORCH-1 | TORCH | Not reported | Phase 3 | Open-label | Yes | PFS and OS |
| 4-WJTOG3405-1 | WJTOG3405 | UMIN 000000539 | Phase 3 | Open-label | Not reported | PFS and OS |
| 4-Yu 2014-1 | Yu 2014 | NCT01769066 | Phase 2 | Open-label | Yes | PFS and OS |
| 5-Butts 2014-1 | START Trial | NCT00409188 | Phase 3 | Blinded | Not reported | PFS and OS |
| 5-Fujisawa 1996-1 | Fujisawa 1996 | Not reported | Phase 2 | Open-label | Not reported | OS |
| 5-Giovanni 1996-1 | Giovanni 1996 | Not reported | Not reported | Open-label | Not reported | OS |
| 5-Katakami 2017-1 | Katakami 2017 | NCT00960115 | Other | Blinded | Not reported | PFS and OS |
| 5-Macchiarini 1991-1 | Macchiarini 1991 | Not reported | Not reported | Open-label | Not reported | PFS and OS |
| 5-Matthay 1986-1 | Matthay 1986 | Not reported | Not reported | Open-label | Not reported | OS |
| 5-Multhoff 2020-1 | Multhoff 2020 | Not reported | Phase 2 | Open-label | Not reported | PFS |
| 5-Stanley 1986-1 | Stanley 1986 | Not reported | Not reported | Not reported | Not reported | OS |
| 5-Vansteenkiste 2013-1 | Vansteenkiste 2013 | NCT00290355 | Phase 2 | Not reported | Not reported | PFS and OS |
| 5-Vansteenkiste 2016-1 | Vansteenkiste 2016 | NCT00480025 | Phase 3 | Blinded | Not reported | PFS and OS |
| 5-Zhao 2014-1 | Zhao 2014 | Not reported | Not reported | Not reported | Not reported | PFS |
| 6-Guo 2020-1 | Guo 2020 | ChiCTR1900024143 | Phase 2 | Not reported | Not reported | PFS and OS |
| 6-Monk 2010-1 | Monk 2010 | Not reported | Phase 2 | Open-label | Not reported | PFS and OS |
| 6-Symonds 2015-1 | Symonds 2015/CIRCCa | ISRCTN23516549 | Phase 2 | Blinded | Not reported | PFS and OS |
| 6-Tewari 2014-1 | Tewari 2014 | NCT00803062 | Phase 3 | Open-label | Not reported | PFS and OS |
| 7-Carbone 2017-1 | CheckMate 026 | NCT02041533 | Phase 3 | Open-label | Yes | PFS and OS |
| 7-Hellmann 2018-1 | CheckMate 227 | NCT02477826 | Phase 3 | Open-label | Yes | PFS and OS |
| 7-Herbst 2020-1 | Herbst 2020 | NCT02409342 | Phase 3 | Open-label | Yes | PFS and OS |
| 7-Mok 2019-1 | Mok 2019 - KEYNOTE-042 | NCT02220894 | Phase 3 | Open-label | Yes | PFS and OS |
| 7-Reck 2016-1 | Reck 2016 | NCT02142738 | Phase 3 | Open-label | Yes | PFS and OS |
| 7-Rizvi 2020-1 | MYSTIC | NCT02453282 | Phase 3 | Open-label | Yes | PFS and OS |
| 7-Sezer 2020-2 | EMPOWER-Lung 1 | NCT03088540 | Phase 3 | Open-label | Yes | PFS and OS |
| 8-Bellmunt 2017-1 | Bellmunt 2017 | NCT02256436 | Phase 3 | Open-label | Yes | PFS and OS |
| 8-Galsky 2020-1 | IMvigor130 | NCT02807636 | Phase 3 | Other | Yes | PFS and OS |
| 8-Powles 2017-1 | IMvigor211 | NCT02302807 | Phase 3 | Open-label | Yes | PFS and OS |
| 8-Powles 2020-1 | DANUBE | NCT02516241 | Phase 3 | Open-label | Yes | PFS and OS |
| 8-Powles 2021-1 | KEYNOTE-361 | NCT02853305 | Phase 3 | Open-label | Yes | PFS and OS |
| PFS: Progression-free survival; OS: Overall survival | | | | | | |
